# Unravelling Cellular Mechanisms of Stem Cell Senescence: An Aid from Natural Bioactive Molecules

**DOI:** 10.3390/biology9030057

**Published:** 2020-03-20

**Authors:** Sara Cruciani, Giuseppe Garroni, Giorgio Carlo Ginesu, Angela Fadda, Carlo Ventura, Margherita Maioli

**Affiliations:** 1 Department of Biomedical Sciences, University of Sassari, Viale San Pietro 43/B, 07100 Sassari, Italy; sara.cruciani@outlook.com (S.C.); giugarroni21@gmail.com (G.G.); 2 General Surgery Unit 2 “Clinica Chirurgica”, Department of Medical, Surgical and Experimental Sciences, University of Sassari, Viale San Pietro 8, 07100 Sassari, Italy; ginesugc@uniss.it; 3 Instituto di Scienze delle Produzioni Alimentari (ISPA), Consiglio Nazionale delle Ricerche (CNR), Traversa la Crucca 3, 07100 Sassari, Italy; angela.fadda@cnr.it; 4 Laboratory of Molecular Biology and Stem Cell Engineering, National Institute of Biostructures and Biosystems–Eldor Lab, Innovation Accelerator, Consiglio Nazionale delle Ricerche, 40129 Bologna, Italy; ventura.vid@gmail.com; 5 Department of Biomedical Sciences, Center for Developmental Biology and Reprogramming (CEDEBIOR), University of Sassari, Viale San Pietro 43/B, 07100 Sassari, Italy; 6 Istituto di Ricerca Genetica e Biomedica, Consiglio Nazionale delle Ricerche (CNR), Monserrato, 09042 Cagliari, Italy

**Keywords:** cellular mechanisms, senescence, stem cells, nutraceuticals, gene expression, oxidative stress

## Abstract

Cellular senescence plays a role in the onset of age-related pathologies and in the loss of tissue homeostasis. Natural compounds of food or plants exert an important antioxidant activity, counteracting the formation of harmful free radicals. In the presence of an intense stressing event, cells activate specific responses to counteract senescence or cell death. In the present paper, we aimed at evaluating the levels of expression of specific markers of senescence, in order to demonstrate that extracts from *Myrtus Communis L*. can prevent premature senescence in ADSCs exposed to oxidative stress. Cells were cultured in the presence of *Myrtus* extracts for 12–24 and 48 h and then incubated with H_2_O_2_ to induce senescence. We then evaluated the expression of senescence-related markers p16, p19, p21, p53, TERT, c-Myc, and the senescence-associated β-Galactoidase activity. Our results showed that pre-treatment with *Myrtus* extracts protects cells from premature senescence, by regulating the cell cycle, and inducing the expression of TERT and c-Myc. These findings suggest a potential application of these natural compounds in the prevention and treatment of various diseases, counteracting premature senescence and preserving tissue functions.

## 1. Introduction

Aging is a complex cellular response to stress. The accumulation of senescent cells in tissues contributes to the loss of homeostasis and the onset of age-related pathologies [1]. The generation of reactive oxygen species (ROS) is essential to preserve tissue integrity. However, an altered balancing in ROS production is related to several diseases and cellular damages [2]. Natural molecules present in food or plants are largely known for their antioxidant activity, counteracting the formation of free radicals through the inhibition of lipid oxidation [3]. Moreover, these small molecules can be used as an adjuvant treatment beside conventional therapy in cancer, due to their ability to reduce cancer cell viability without affecting the proliferation of normal healthy cells [4,5,6]. *Myrtus communis* L. has a large amount of galloyl derivatives, as flavonols, and tannins in its berries, seeds and leaves, exerting important antioxidant and antimutagenic activities [7,8]. In the presence of an intense stressor, cells activate specific responses and mechanisms of repair, in order to counteract senescence or cell death [9,10]. Oxidative stress can induce changes in DNA and RNA structure, promoting senescence in mesenchymal stem cells (MSCs) aging, thus affecting their functions and longevity [11,12]. Excessive accumulation of ROS can induce a dysregulation of stem cell migration and differentiation potential [13] related to the suppression of cell growth, the altered expression of specific markers of senescence and a reduced activity of telomerase (TERT) [14,15]. Within this context, an interesting hypothesis relates stem cell senescence and their diminished regenerative potential to ageing-related diseases [12]. The expression of cyclin-dependent kinase (CDK) inhibitors p16INK4A, p19ARF, p21 and cell cycle arrest are closely associated with senescence, as well as the increased activity of β-galactosidase [16,17]. DNA damage or other stressful stimuli can also induce the activation of p53, promoting cell-cycle arrest or apoptosis [18,19]. In addition, the down-regulation of c-Myc is related to a pro-inflammatory senescent phenotype, vascular aging and endothelial dysfunction-associated pathologies [20]. Within this context, we have previously demonstrated that residues from myrtle liqueur production were able to counteract the appearance of a senescent phenotype in adipose-derived stem cells (ADSCs), exposed to H_2_O_2_. Moreover, they were also able to implement their regenerative potential, modulating the expression of stemness-related genes. In the present paper, we aimed at investigating a gene program responsible for cell senescence in human adipose-derived stem cells (hADSCs) exposed to oxidative stress, eventually modulated by our extracts, thus disclosing novel natural resources for future clinical applications.

## 2. Materials and Methods

The laboratory and industrial biomasses (by-products) of myrtle (Lab by-P and Ind by-P) used in this study, were collected and freeze-dried as previously described [19,20], and then used in vitro on hADSCs. hADSCs, isolated after written informed consent from human adult subcutaneous adipose tissue [21,22], were cultured in a basic growing Dulbecco’s modified Eagle’s Medium (DMEM, Life Technologies, Carlsbad, CA, USA), supplemented with 20% fetal bovine serum (FBS, Life Technologies, Carlsbad, CA, USA), 200 mM L-glutamine (Euroclone, Milano, Italy), and 200 U/mL penicillin −0.1 mg/mL streptomycin (Euroclone, Milano, Italy). Cells at passage 5 were then cultured in the presence of 0.5 mg/mL *Myrtus* extracts for 12–24 and 48 h. Untreated control cells were cultured in the basic growing medium alone. At the end of the incubation time, cells were incubated with 100 μM H_2_O_2_ for 1 h, to induce senescence. Positive control was represented by hADSCs cultured in the presence of 100 μg/mL ascorbic acid (Sigma-Aldrich, Darmstadt, Germany), and then senescence was induced. To perform gene expression analysis, total RNA was isolated from cells treated in different described conditions at times 0, 12, 24, and 48 h, using Trizol reagent (Life Technologies, Carlsbad, CA, USA), according to manufacturer’s instructions. About 1 μg of total RNA was reverse-transcribed into cDNA using the Superscript Vilo cDNA synthesis kit (Life Technologies, Carlsbad, CA, USA) and used for the quantitative polymerase chain reaction. qRT-PCR was performed in triplicate under standard conditions, according to the protocol specified in the Platinum^®^ Quantitative PCR SuperMix-UDG Kit, using a CFX Thermal Cycler (Bio-Rad) (Applied Biosystems, Foster City, CA, USA). The mRNA levels of hADSCs treated in different conditions were expressed as fold of change (2^−∆∆Ct^) relative to the mRNA levels observed in hADSCs at time 0, before starting the treatment. Target Ct values were normalized on HPRT1, considered as a reference gene. The genes analyzed were: p16, p19, p21, p53, TERT and c-Myc. All primers used were from Invitrogen and previously described [21]. For each treatment, three technical replicates were performed twice and analyzed using Statistical Package for the Social Sciences version 13 Software (SPSS Inc., Chicago, IL, USA). The distributions of variance of each group were evaluated with Kruskal–Wallis rank sum and Wilcoxon signed-rank test, assuming a *p* value < 0.05 as statistically significant.

To evaluate senescent-associated β-Galactoidase activity on fibroblast induced to oxidative stress, “The Senescence Cells Histochemical Staining Kit” (Sigma-Aldrich, Darmstadt, Germany) was used. ADSCs are cultured in the presence or absence of *Myrtus* extracts and then senescence was induced. At the end of the incubation time, the medium containing H_2_O_2_ was removed; cells were then fixed and processed according to the manufacturer’s instructions. The number of positively blue-stained cells was calculated as the percentage of the total number of cells.

## 3. Results

**Figure 1:**
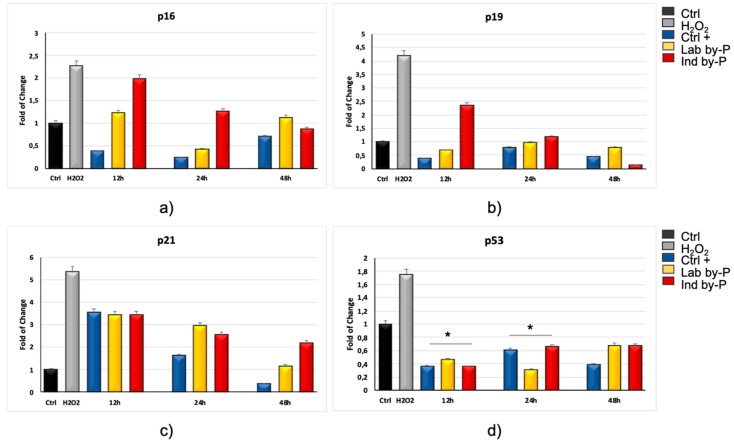
Expression of specific markers of senescence p16INK4A, p19ARF, p21 and p53. The expression of p16INK4A (**a**), p19ARF (**b**), p21 (**c**) and p53 (**d**) was evaluated in H_2_O_2_-senescent hADSCs pre-exposed for 12, 24, or 48 h to ascorbic acid (CTRL+, blue bars) or to Lab by-P (yellow bars) or Ind by-P (red bars). Grey bars represent the hADSCs exposed to H_2_O_2_, without pre-treatment with the different compounds. The mRNA levels for each gene were expressed as fold of change (2^−∆∆Ct^) of mRNA levels observed in untreated hADSCs (CTRL-, black bar) defined as 1 (mean ± SD; *n* = 6) and normalized to hypoxanthine phosphoribosyltransferase 1 (HPRT1). Data are represented as mean ± SD referring to the control (* *p* ≤ 0.05).

Figure 1 shows that *Myrtus* extracts are able to prevent senescence in hADSCs pre-treated with by-products for 12, 24, or 48 h, and then exposed to H_2_O_2_. In particular, in these cells the expression of p16 (Figure 1a), p19 (Figure 1b), p21 (Figure 1c) and p53 (Figure 1d) and decreased after 12 h of treatment, as compared to cells exposed to H_2_O_2_ alone (grey bars), in all the experimental conditions, being superimposable to the mRNA levels of control untreated cells (black bars). In hADSCs exposed to *Myrtus* extracts for 12 or 24 h, p53 gene expression was even significantly lower than the transcriptional level observed in control untreated cells (Figure 1d).

**Figure 2:**
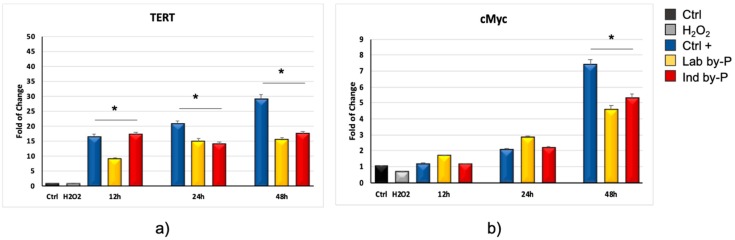
Expression of specific markers of senescence TERT and c-Myc. The expression of TERT (**a**), and c-Myc (**b**) was evaluated in H_2_O_2_-senescent hADSCs exposed for 12, 24, or 48 h to ascorbic acid (CTRL+, blue bars) or to Lab by-P (yellow bars) or Ind by-P (red bars). Grey bars represent the hADSCs exposed to H_2_O_2_, without pre-treatment with the extracts. The mRNA levels for each gene were expressed as fold of change (2^−∆∆Ct^) of mRNA levels observed in untreated hADSCs (CTRL-, black bar) defined as 1 (mean ± SD; *n* = 6) and normalized to hypoxanthine phosphoribosyltransferase 1 (HPRT1). Data are represented as mean ± SD referring to the control (* *p* ≤ 0.05).

In cells hADSCs pre-treated with the by-products for 12, 24, or 48 h and then exposed to H_2_O_2._ The mRNA levels of TERT were significantly upregulated, starting from 12 h of exposure (Figure 2a). In the same ADSC pre-treated with the extracts, even c-Myc was upregulated, as compared to control untreated cells, reaching a maximum after 48 h of treatment (Figure 2b).

**Figure 3:**
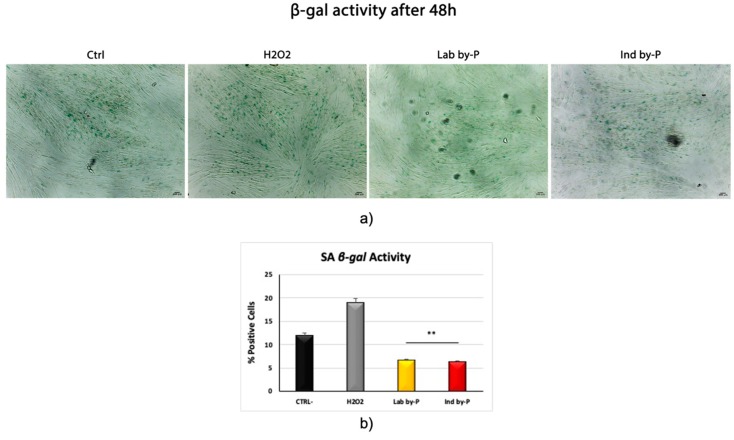
Senescence-associated β-galactosidase activity. (**a**) β-galactosidase was evaluated in H_2_O_2_-senescent ADSCs treated with Lab by-P, or with Ind by-P, compared to control untreated ADSCs (Ctrl). Scale bar = 100 μm. (**b**) The numbers of positive (blue) and negative cells were counted under the light microscope and the percentage of SA-β-Gal-positive cells for each treatment was calculated as the number of positive cells divided by the total number of cells counted using an image software analysis (ImageJ). Data are expressed as mean ± SD referring to the control (* *p* ≤ 0.05; ** *p* ≤ 0.01).

Figure 3 shows β-galactosidase activity in ADSCs, treated with *Myrtus* by-Products and then exposed to H_2_O_2_. Both Lab by-P and Ind by-P extracts were able to significantly counteract the number of blue-stained premature senescent cells, as compared to both control untreated cells, or to cells exposed to H_2_O_2_ without extracts pre-treatment.

## 4. Discussion and Conclusions

ROS have been recognized as toxic and harmful by-products for stem cells, resulting in DNA damage, premature senescence or cell death [22]. MSCs are largely known for their ability to restore tissue function and differentiate into several phenotypes under appropriate stimuli [23], including osteogenic, chondrogenic, cardiogenic and adipogenic lineages [24,25,26,27]. Nevertheless, as previously demonstrated by Liao N et al., hADSCs expanded for different passages, showing high mRNA levels of p16, p21 and p53 and an increased ROS production, associated with the appearance of a senescent phenotype. Within this context, antioxidants may be used to preserve stemness, reducing ROS production and aging [28]. An imbalance in ROS homeostasis induces premature cellular senescence, telomere shortening and a related reduced ability of MSCs to differentiate along multiple lineages [29,30]. It is largely recognized that senescent MSCs secrete several factors that induce a state of inflammation at a systemic level, responsible for decreasing the migratory and immunomodulation activities of cells [13]. The expression of telomerase declines with age, and in addition senescent stem cells show a low activity of this enzyme, leading to telomere shortening and the increased secretion of pro-inflammatory cytokines, influencing the behavior of the neighboring cells [31]. For these reasons, researchers in recent years have been trying to dissect the epigenetic modifications involved in cellular aging, in an attempt to reverse stem cell senescence and achieve rejuvenation [32,33]. Physical stimuli such as electromagnetic fields have been largely applied to slow down cell senescence, by modulating cell polarity and the expression of the aging related markers, such as TERT, p16INK4, ARF, p53, and p21, together with the activity of beta-galactosidase [21,34,35,36]. Moreover, other authors demonstrated that Rapamycin can inhibit mTOR and its signalling pathway, restoring both the self-renewal and the potency of stem cells [37]. Natural or synthetic molecules have previously been shown to be capable of inducing growth arrest in cancer cells, while restoring cellular homeostasis [38]. Plants contain a large number of compounds in their leaves, berries and seeds, exerting an important antioxidant activity, counteracting ROS accumulation and aging [39,40]. The present paper aimed at evaluating the protective and anti-senescent activity of *Myrtus* pulp and seed extracts in senescence-induced hADSCs. In previous published papers, we demonstrated a role of these natural compounds, acting through epigenetic mechanisms, in counteracting inflammation, oxidative stress and ROS accumulation in cells exposed to a strong stressing condition [41,42]. In the present study, we show that pre-treatment with *Myrtus* extracts protects human stem cells from premature senescence, with a significant downregulation of the master gene regulators of cell cycle, concomitantly increasing the expression of TERT and c-Myc. Taken together, our results indicate a protective role of the investigated extracts against oxidative stress, preserving telomerase expression and cell longevity, thus suggesting a potential application of these natural compounds in preventing and managing various senescence-associated diseases. In particular, the observation that extracts were effective in counteracting the upregulation of senescence-associated gene expression supports the future clinical application of these compounds as potential tools for blunting age-related degenerative diseases. Nevertheless, other studies are needed in order to translate the results obtained in vitro into novel therapeutic therapies for regenerative medicine.
